# High-quality genome assembly and annotation of the crested gecko (*Correlophus ciliatus*)

**DOI:** 10.1093/g3journal/jkae265

**Published:** 2024-11-15

**Authors:** Ruyi Huang, Jinghang Zhang, Liang Lu, Song Huang, Chenhong Li

**Affiliations:** Shanghai Universities Key Laboratory of Marine Animal Taxonomy and Evolution, Shanghai Ocean University, Shanghai 201306, China; Anhui Province Key Laboratory of the Conservation and Exploitation of Biological Resource, College of Life Sciences, Anhui Normal University, Wuhu 241000, China; Engineering Research Center of Environmental DNA and Ecological Water Health Assessment, Shanghai Ocean University, Shanghai 201306, China; Shanghai Universities Key Laboratory of Marine Animal Taxonomy and Evolution, Shanghai Ocean University, Shanghai 201306, China; Engineering Research Center of Environmental DNA and Ecological Water Health Assessment, Shanghai Ocean University, Shanghai 201306, China; Shanghai Universities Key Laboratory of Marine Animal Taxonomy and Evolution, Shanghai Ocean University, Shanghai 201306, China; Engineering Research Center of Environmental DNA and Ecological Water Health Assessment, Shanghai Ocean University, Shanghai 201306, China; Anhui Province Key Laboratory of the Conservation and Exploitation of Biological Resource, College of Life Sciences, Anhui Normal University, Wuhu 241000, China; Shanghai Universities Key Laboratory of Marine Animal Taxonomy and Evolution, Shanghai Ocean University, Shanghai 201306, China; Engineering Research Center of Environmental DNA and Ecological Water Health Assessment, Shanghai Ocean University, Shanghai 201306, China

**Keywords:** Gekkonoidea, genome, chromosome, Nanopore, Hi-C

## Abstract

*Correlophus ciliatus*, or the crested gecko, is widely kept as a pet in many countries around the world due to its ease to care and bred and its high survival rate. However, there is limited number of genomic studies on the crested gecko. In this study, we generated a high-quality chromosome-level genome assembly of the crested gecko by combining Nanopore, Illumina, and Hi-C data. The genome assemble has a size of 1.66 Gb, with scaffold N50 of 109.97 Mb, and 99.52% of the scaffold anchored on 19 chromosomes. The BUSCO analysis indicated a gene completeness of 90.3% (*n* = 7,480), including 6,673 (89.2%) single-copy genes and 84 (1.1%) duplicated genes. Additionally, we identified 21,065 protein-coding genes using the MAKER3 annotation toolkit, with 41.98% (697.51 Mb) consisting of repetitive elements. Among these, 21,037 genes were validated through InterProScan5. Our study is the first to report a chromosome-level genome for the crested gecko. It provides valuable genomic resources for understanding molecular mechanisms under many interesting traits of the species.

## Teaser

Huang *et al*. report a high-quality genome assembly and annotation of the crested gecko, obtained using Illumina, Nanopore, and Hi-C data, with annotated key genomic elements. The genome assembly has a size of 1.66 GB, with scaffold N50 of 109.97 MB, and 99.52% of the scaffold anchored on 19 chromosomes.

## Introduction

*Correlophus ciliatus* (Guichenot 1866), or the crested gecko, is endemic to New Caledonia, found in scattered forested areas at elevations between 150 and 1,000 m. Its distribution range is estimated to be 5,861 km^2^, and the area of occupancy is estimated to be 44 km^2^. Currently classified as Vulnerable by the IUCN (International Union for Conservation of Nature, Sadlier *et al*. 2021), this species faces a problem of restricted distribution and ongoing declines due to degradation of habitat quality, and at least 50% of its range is likely under threatened by habitat loss and fragmentation.

The crested gecko is a nocturnal species that rests on tree branches during the day and becomes active at night, moving through the high canopy of tropical rainforests. It skillfully jumps between branches while foraging for food and water. Unlike many ground-dwelling lizards, it thrives in the upper forest layers and has an omnivorous diet, feeding on small invertebrates like insects as well as fruits, setting it apart from strictly insectivorous or herbivorous species. Due to its adorable appearance and unique lifestyle, the crested gecko has become popular among reptile enthusiasts and researchers. Achieving widespread artificial domestication and breeding in under 20 years is a rare phenomenon among reptiles. Few reptile species are amenable to genetic editing, particularly those that reproduce oviparous. In 2019, the results of CRISPR-Cas9 gene editing experiments on unfertilized *Anolis sagrei* oocytes were published (Rasys *et al*. 2019). *C. ciliatus*, a pad-bearing arboreal species that breeds prolifically in captivity, is suitable for similar CRISPR-Cas9 gene editing experiments to verify some interesting biological phenomena in arboreal lizards of this kind.

Genomic data in squamate reptiles (lizards and snakes) has lagged behind other vertebrate model systems, such as birds and mammals, and high-quality reference genomes remain scarce (Pinto *et al.* 2023). Of the 23 previously published chromosome-scale squamate reference genomes, only 12 of the ∼60 families are represented. Within geckos (infraorder Gekkota), a species-rich clade of lizards, chromosome-level genomes are exceptionally sparse representing only 3 (Eublepharidae, Gekkonidae, Sphaerodactylidae) of the 7 extant families.

A complete genome assembly is crucial for understanding the molecular mechanism underlying its interesting biological traits. In this study, we obtained a high-quality genome of the crested gecko using Illumina, Nanopore, and Hi-C data, and annotated key genomic elements including repeat elements and protein-coding genes. This work provides valuable genomic data for studying molecular mechanisms and evolution of the gecko species.

## Materials and methods

### Sample preparation

**Fig. 1.**
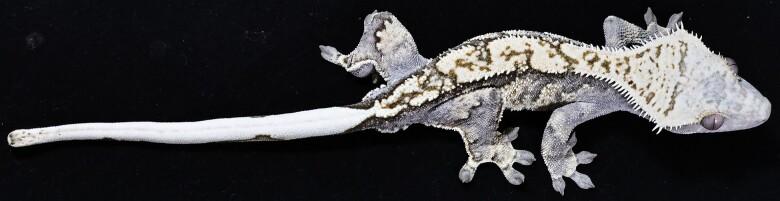
The specimen of the crested gecko (*Correlophus ciliatus*), adult male.

A single adult male crested gecko (Fig. 1) was acquired from a Chinese pet trade store and euthanized with MS-222 (Beaupre *et al*. 2004). Genomic DNA was extracted from muscle tissue using the Qiagen Tissue kit (DP304-03). Liver samples were collected from the same gecko and immediately preserved in liquid nitrogen for 3D conformation analysis. Total RNA was extracted from 11 tissues (muscle, heart, skin, liver, hemipenis, lung, spine, tone, seminal vesicle, eye, and brain) using the standard TRIzol protocol (Rokyta *et al*. 2012), and RNA was isolated using the TRlzol reagent (Qiagen RNeasy fibrous tissue mini kit). The specimen is vouched in the Collection of Shanghai Ocean University, Shanghai, China (voucher code: SOU1801018; contact person: Dr Ya Zhang, email: zhangya@shou.edu.cn).

### Library preparation and sequencing

The extracted DNA from muscle tissue was purified for large fragments using magnetic beads and inspected with pulsed-field gel electrophoresis and NanoDrop One (Thermo Fisher Scientific, Lafayette, USA). Genomic DNA was used for both short-read and long-read whole genome sequencing. Libraries with an insert size of 350 bp were constructed for short-read DNA sequencing and transcriptome sequencing using an insert size of 350 bp library following the Illumina Sequencing Library Preparation protocol (Meyer and Kircher 2010) and using the NEBNext Ultra RNA Library Prep Kit [New England Biolabs (NEB), Ipswich, USA], respectively. The libraries were sequenced on an Illumina NovaSeq 6000 platform (Novagene, Beijing, China).

Large-sized fragment libraries were prepared using the ONT Template Prep Kit [SQK-LSK109, Oxford Nanopore Technologies (ONT), Oxford, UK]. DNA fragments were end-repaired and 3′-adenylated with the NEBNext FFPE DNA Repair Mix kit (NEB, Ipswich, USA). Nanopore sequencing adapters were ligated using the NEBNext Quick Ligation Module (E6056) (NEB). The final library was sequenced on the Nanopore PromethION platform (ONT).

The Hi-C library was constructed using nuclear DNA from liver tissue cells, which were cross-linked and digested with the restriction endonuclease DpnII. DNA fragments with interaction relationships were captured using streptavidin beads and prepared for sequencing. The final Hi-C library was sequenced on an Illumina HiSeq 2500 platform to obtain 2 × 150 paired-end reads (Novagene, Beijing, China).

### Genome size estimation and assembly

The raw reads were subjected to quality control using fastp v0.21.0 (Chen *et al*. 2018) to filter adapter sequences and low-quality reads with default parameter settings. The filtered reads were then used to analyze the 21-mer distribution using Jellyfish v2.2.10 (Marçais and Kingsford 2011) and estimate the genome size of the crested gecko by using GenomeScope v2.0 (Vurture *et al*. 2017).

The draft genome was obtained by first assembling long reads and then polishing the results with short reads, which has been widely used in recent genome assembly for different organisms (Shu *et al*. 2023). NextDenovo v2.5.0 (Hu *et al*. 2024) was used to assemble the ONT sequences with the default parameters and “genome_size = 1451744020, parallel_jobs = 5”. NextPolish v1.4.0 (Hu *et al*. 2020) was then applied to polish the draft genome assembly using NGS sequences with the default parameters. Purge Haplotigs (Roach *et al*. 2018) was used to extract genomic haplotypes and removed the redundant data. Benchmarking Universal Single-copy Orthologs (BUSCO) v5.5.0 with the “sauropsida_odb10” database was used to examine the completeness of the final assembled genome (Simão *et al*. 2015) with options “-m geno -c 20 -1 sauropsida_odb10 --augustus”.

BWA v0.7.17 (Li 2013) was used to align Hi-C reads, and Juicer v1.6.2 (Durand *et al.* 2016b) was used to quality control with the default parameters and obtain the interaction matrix. 3D-DNA v180922 (Dudchenko *et al*. 2017) was used to anchor the primary contigs onto chromosomes (options -r 6), then possible errors were corrected manually with Juicebox v1.11.08 (Durand *et al.* 2016a).

### Genome synteny analysis

The synteny of the crested gecko assembly was compared to that of the Townsend's dwarf gecko (*Sphaerodactylus townsendi*), both of which are members of the same superfamily Pygopodidae. Chromosome synteny between the crested gecko and the Townsend's dwarf gecko was detected by MCScanX (Wang *et al*. 2012) with default parameters. The genome assembly of the Townsend's dwarf gecko (Pinto *et al*. 2022) was retrieved from NCBI with accession number GCA_021028975.2. The visual diagram was generated using AdobeIllustrator2021.

### Gene prediction and annotation

The genome of the crested gecko was predicted for repeat elements using RepeatModeler v2.0.3 (Arian and Robert 2008) with default settings. The database of repeat elements constructed using RepeatModeler contains all repeated elements, which may also contain coding sequences (CDS), so the repeat elements were aligned to CDS of geckos and lizards using BLAST (Zhang *et al*. 2000) to verify the database of repeat elements. The genome was then screened for repetitive elements with RepeatMasker v4.1.2 (Arian and Robert 2013) based on the constructed repeat elements database. Gene prediction was carried out using the Maker v3.01.04 pipeline (Cantarel *et al*. 2008), which employed both de novo and homology-based methods. Augustus v3.3 (Stanke and Waack 2003), integrated into Maker, was used to predict de novo genes, trained with *Gallus gallus* (-augustus_species = chicken). All geckos and lizards’ genes were retrieved from the NCBI database using the “Taxonomy” option (Lepidosauria/Taxonomy ID: 8504) for reference in gene prediction. Additionally, transcriptome data of the crested gecko, obtained from our own sequenced samples across 11 tissues, were used for annotation. The transcriptome was mapped to the genome using Blastn v2.7.1 (est2genome parameter in Maker). These gene models were used to train the hidden Markov models of Augustus and SNAP (Korf 2004) for iterative genome annotation, which was repeated for 2 extra times. MAKER3 was used to identify alternative splice forms where EST data allowed, provided quality control metrics for each gene model, and selected the best-fitting gene model. To filter the predicted genes, we refined our data according to AED score (annotation edit distance). The gene with AED score < 0.5 was retained. Finally, well-annotated databases InterProScan 5.69-101.0 (Jones *et al*. 2014) were applied to conduct the functional annotation. In this process, all the parameters were set as default and the mainstream databases including Pfam, Gene3D, SUPERFAMILY, Smart, CDD, and Coils databases in InterProscan5 were used for search. Finally, The Infernal (Nawrocki and Eddy 2013) cmscan v1.1.4 program was utilized to identify micro-RNAs, ribosomal RNAs (rRNAs), small nuclear RNAs, and small nucleolar RNAs by conducting a search against the Rfam database.

### Phylogenetic analysis

OrthoFinder v2.4.0 (Emms and Kelly 2019) was used to retrieve the orthologous genes of 15 squamate genomes, including *Eublepharis macularius* (assembly accession: GCF_028583425.1), *Euleptes europaea* (assembly accession: GCF_029931775.1), *Sphaerodactylus townsendi* (assembly accession: GCF_021028975.2), *Paroedura picta* (assembly accession: GCA_003118565.2), *Gekko japonicus* (assembly accession: GCF_001447785.1), *Heteronotia binoei* (assembly accession: GCF_032191835.1), *Hemicordylus capensis* (assembly accession: GCF_027244095.1), *Podarcis muralis* (assembly accession: GCF_004329235.1), *Varanus komodoensis* (assembly accession: GCF_004798865.1), *Candoia aspera* (assembly accession: GCF_035149785.1), *Python bivittatus* (assembly accession: GCF_000186305.1), *Thamnophis sirtalis* (assembly accession: GCF_001077635.1), *Ahaetulla prasina* (assembly accession: GCF_028640845.1), and *Pantherophis guttatus* (assembly accession: GCF_029531705.1), and 1 turtle species *Chrysemys picta bellii* (assembly accession: GCF_011386835.1) was selected as an outgroup.

Phylogenetic trees were reconstructed based on single-copy orthologous gens. All single-copy sequences were concatenated into 1 data matrix after being aligned with MAFFT v7.505 (Katoh and Standley 2013). The phylogenetic tree was constructed using IQ-TREE v2.1.4 (Minh *et al*. 2020) with the best model (bird + F + R10) estimated by ModelFinder (Kalyaanamoorthy *et al*. 2017). Statistical support for the phylogenetic trees was evaluated by Ultrafast bootstrap (Hoang *et al*. 2018) analysis using 1,000 replicates to assess the robustness of the tree.

### Population history analysis

Pairwise sequentially Markovian coalescent (PSMC) analysis (Li and Durbin 2011) was performed to infer the population size history of crested gecko. The analysis used a diploid consensus sequence generated from whole genome data. The 80× Illumina short polishing reads were mapped to the assembled genome of crested gecko using the “BWA-MEM” algorithm from BWA v0.7.17 (Li 2013) with the parameter “mem -t 30” and input the assembled genome and NGS sequencing data. SAMtools v0.1.19 (Li *et al*. 2009) was then employed to produce the diploid consensus, applying default settings except for the parameters “-d 26 -D 160”. The PSMC analysis adopted default settings, with a generation time of 2 years and a substitution rate set at 5.6 × 10⁻^10^ per site per year, based on data from squamate reptiles (Perry *et al*. 2018).

## Results and discussion

### Sequencing results and k-mer analysis

The total data generated from the long-read sequencing was 137.55 Gb, while the total data generated from the short-read sequencing was 412.39 Gb (Table 1).

**Table 1.** Statistics of the DNA/RNA sequencing data used for genome assembly of the crested gecko.

Library	Inset size (bp)	Reads number	Raw data (Gb)	N50 read length (bp)	Average coverage (×)
Illumina	350	923,968,260	135.86	—	81.83
ONT	20,000	5,341,769	137.55	33,226	82.86
Hi-C	350	670,448,741	206.32	—	124.29
RNA-seq	350	809,620,078	70.21	—	—
Total	—	2,409,378,848	549.94	—	—

**Fig. 2.**
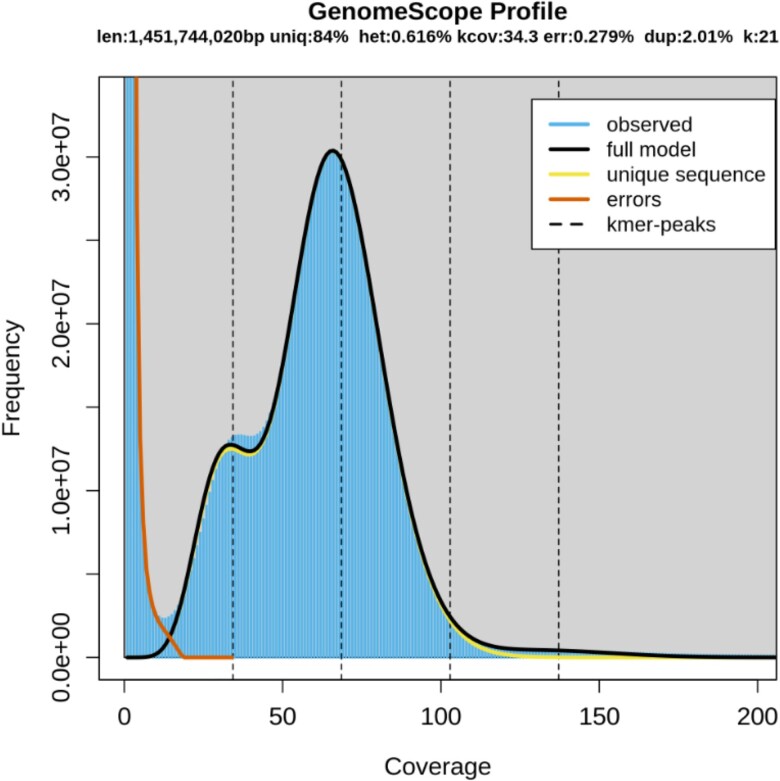
Histogram of the 21-mer depth distribution of the sequencing reads of the crested gecko plotted in GenomeScope.

Before genome assembly, we extracted Illumina paired sequencing reads with approximately 50×coverage to estimate genome size and heterozygosity of the crested gecko. The 21 k-mer frequency spectra obtained with Jellyfish v1.1.10 and GenomeScope suggested that the heterozygosity of the crested gecko genome was 0.616%, the estimated genome size was 1.45 Gb, and the repeat sequence content was 16.0% (Fig. 2).

### Genome assembly and evaluation

**Fig. 3.**
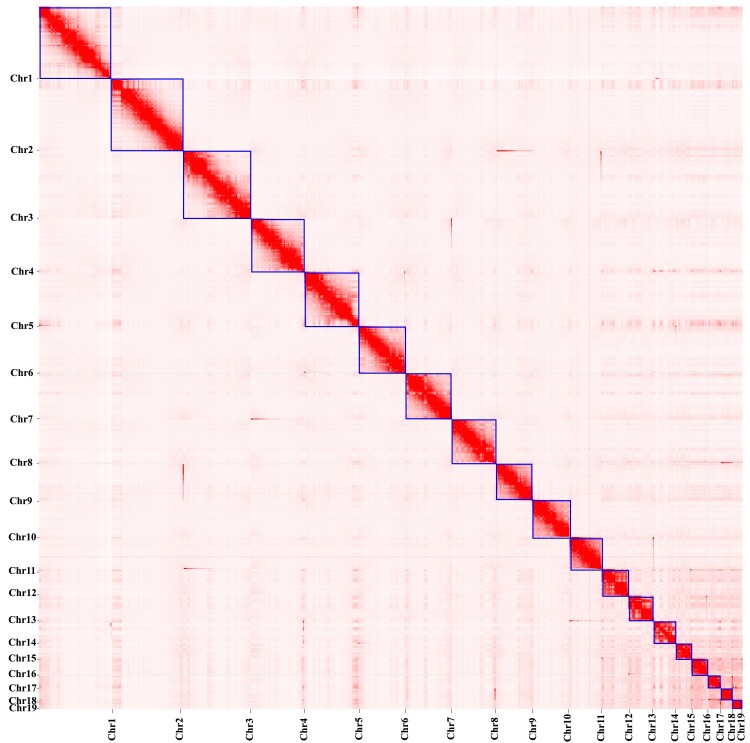
Genome-scale chromosome heatmap of *Correlophus ciliatus* shows 19 individual chromosomes, ordered from longest to shortest, outlined in squares.

The final genome assembly of the crested gecko was 1.66 Gb with a scaffold N50 of 109.97 Mb. We extracted scaffold fragments that were not assembled into chromosomes and uploaded them to NCBI for BLAST comparison, and checked the assembled scaffolds for contamination and excluded 9 scaffolds (scaffold 8, scaffold 4, scaffold 38, scaffold 40, scaffold 45, scaffold 53, scaffold 68, scaffold 134, and scaffold 198) potentially originating from *Homo sapiens*, *Gorilla gorilla*, *Colius striatus*, etc. The parameter “-r” is used to specify the number of iterative rounds for misjoin correction (default is 2). We incrementally increased the “-r” parameter in Hi-C from “-r 0”, modifying this value multiple times to analyze the Hi-C data, until we reached “-r 6” where we achieved a very clear chromosome boundary. The Hi-C analyses generate 19 pseudomolecules (Fig. 3), anchoring 99.52% (∼1.66 Gb) of the genome assembly of crested gecko. The average GC content of the crested gecko genome assembly was 45.01% (Table 2).

**Table 2.** Summary statistics of the crested gecko genome assembly.

	Statistics
Contig N50 (bp)	74,935,118
Number of contigs	303
Scaffold N50 (bp)	109,969,818
Number of scaffolds	190
Maximum scaffold (bp)	170,028,000
Genome size (bp)	1,661,508,015
Number of chromosomes	19
GC content (%)	45.01

In the results of BUSCO analysis, a commendable (90.3%) of complete BUSCOs were identified, which included 89.2% of single-copy genes and 1.1% of duplicated BUSCOs (Table 3). In comparison, the genome assembly of *G. japonicus* contained 93.8% complete BUSCOs sequences, and the BUSCO of *S. townsendi* genome from 85.5 to 88.3% after 11 rounds of genome assembly. Both species belong to the infraorder Gekkota. These results demonstrate that the genome assembly of the crested gecko has a high level of completeness and accuracy.

**Table 3.** Statistical result of BUSCO evaluation on the genome assembly.

	Gene number	Percentage (%)
Complete BUSCOs (C)	6757	90.3
Complete and single-copy BUSCOs (S)	6673	89.2
Complete and duplicated BUSCOs (D)	84	1.1
Fragmented BUSCOs (F)	217	2.9
Missing BUSCOs (M)	506	6.8
Total BUSCO groups searched	7480	100

### Genome synteny analysis

**Fig. 4.**
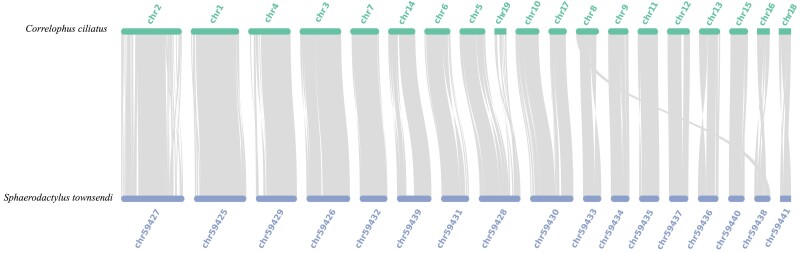
Genome synteny analysis between the crested gecko and the Townsend's dwarf gecko genomes.

The results showed a high level of synteny between the crested gecko and the Townsend's dwarf gecko. Three fusion and fission events were observed between these 2 geckos. Chr5 and chr19 of the crested gecko were syntonic with chr59428 of Townsend's dwarf gecko, while chr10 and chr17 of the crested gecko were syntonic with chr59430 of the Townsend's dwarf gecko. Additionally, chr8 of the crested gecko shows synteny with chr59433 and a portion of chr59438 in the Townsend's dwarf gecko. This indicates evolutionary rearrangements that may have contributed to the divergence of these species (Fig. 4).

### Gene prediction and annotation

We have collected all available tissues, and RNA sequencing data was assembled separately and used as part of the annotation process. There were total 21,065 predicted genes (figshare: 10.6084/m9.figshare.26772169) averaging 1,109 genes per chromosome. We identified a total of 16,702 annotated genes shared among 11 tissues, with 4,363 genes being partially shared. Notably, 22 unique annotated genes were extracted from the transcriptome data of the seminal vesicle. We recommend conducting multiple tissue transcriptome sequencing to obtain more annotation information. Among them, 19,866 genes (94.3%) were successfully annotated using InterProScan5. The BLAST results against various databases are presented in Supplementary File 1.

We found that the crested gecko genome contains 41.98% repeat sequences, with classified DNA transposons comprising 3.06% of the genome. Details of all repeat databases are provided in Supplementary Files 2 and 3. In total, 4,392 elements were identified from noncoding RNAs, comprising 1,267 ribosomal RNAs (rRNAs), 2,656 micro-RNAs (miRNAs), 116 small nuclear RNAs (snRNAs), 34 ribozymes, 219 transfer RNAs (tRNAs), and 100 other RNA types. Among the snRNAs, 81 are spliceosomal RNAs (U1, U2, U4, U5, U6), and 35 are small nucleolar RNAs (snoRNAs). Further details are provided in the Supplementary File 4. The percentage of transcripts with valid functional annotations largely depends on the information available in public databases. All the Software and algorithms used in this article are in Supplementary File 5.

### Results of phylogenetic analysis

**Fig. 5.**
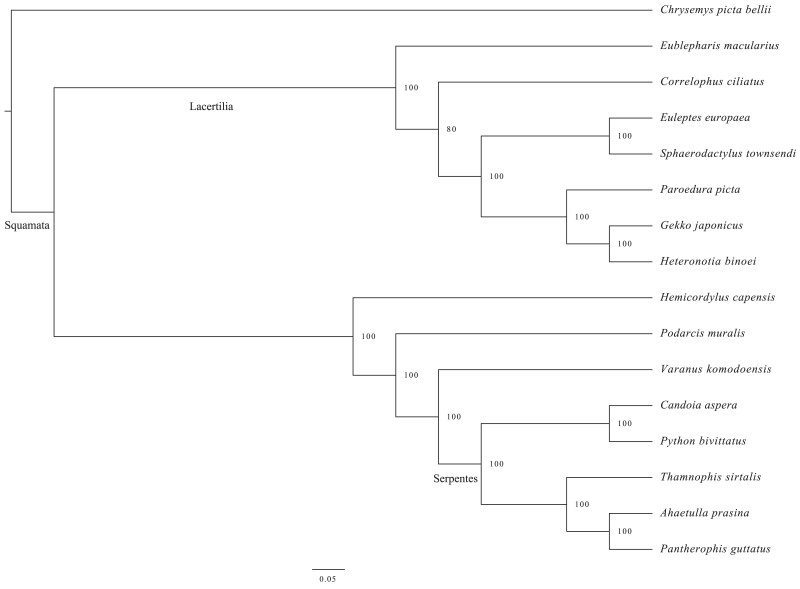
Maximum likelihood tree of the crested gecko and 14 other Squamata species.

The phylogenetic tree reconstructed by using IQ-TREE had high bootstrap support values (Fig. 5). The topology of the phylogeny was consistent with that of previous study (Peng *et al*. 2023).

### Population history

**Fig. 6.**
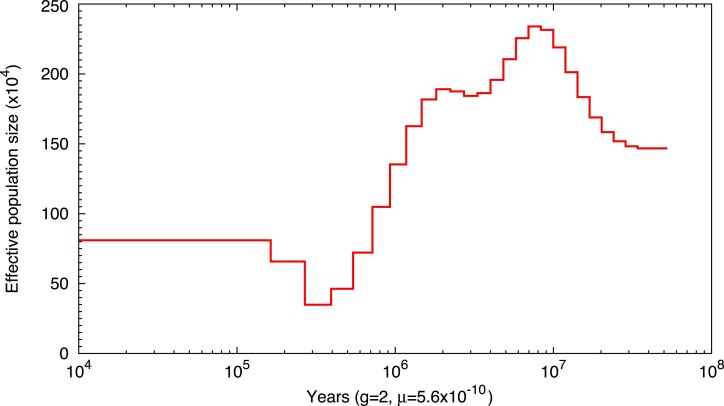
Fluctuation of population size of the crested gecko between 10 Ma to 10 ka estimated using PSMC.

The effective population size of crested gecko between 10 ka and 100 Ma is shown in Fig. 6. From 100 to 10 Ma, the population increased to 2.3 × 10^−6^, possibly due to the fact that during the Late Cretaceous (around 80 Ma), New Caledonia was part of the supercontinent Zealandia. This landmass rifted from Australia, creating an isolated environment that allowed for the evolution of unique species (Kranitz *et al*. 2014). The population then declined continuously, likely due to New Zealand experiencing repeated cycles of major climatic changes, approximately 20 cycles of cooling and warming, starting in the late Pliocene (∼3.0–2.6 Ma) and continuing throughout the Pleistocene (Lee and Forsyth 2008). Around 300 ka, the population began to recover. The expansion during the period of 300–400 ka might align with favorable climatic conditions, and various factors such as sea level fluctuations and volcanic activity could have influenced these dynamics (McDowall 2010). The population reached a stable state around 100 ka.

PSMC has limited temporal resolution for both recent population dynamics (within the last 10,000 years) and distant timescales beyond a few million years (Li and Durbin 2011). As a result, the population size fluctuations inferred for deep time periods (e.g. 100 Ma) may not accurately reflect actual demographic trends and could instead be artifacts stemming from the model's limitations. Moreover, PSMC cannot detect gene flow, meaning that historical admixture or hybridization events with closely related species may have been overlooked, potentially leading to misinterpretations of the timing and magnitude of population changes. Thus, while the general patterns provide useful insights, these results should be interpreted with caution, considering the potential for confounding factors.

## Conclusion

Combining Nanopore, Illumina, and Hi-C technologies, we generated a high-quality chromosome-level genome assembly of the crested gecko. The assembly has a total size of 1.66 Gb, with a scaffold N50 of 109.97 Mb, and 99.52% of the scaffolds anchored to 19 chromosomes. BUSCO analysis revealed a gene completeness of 90.3%, including 89.2% single-copy genes and 1.1% duplicated genes. This is the first chromosome-level genome assembly for the crested gecko, providing an essential resource for studying the molecular basis of many unique traits in this species. With the publication of the crested gecko genome, the representation of chromosome-level genomes within the Gekkota has expanded from only 3 to 4 of the 7 extant families, advancing genomic resources and providing a more comprehensive foundation for evolutionary studies within this lineage.

## Supplementary Material

jkae265_Supplementary_Data

## Data Availability

The raw data are deposited in NCBI with SRA accessions numbers: SRR30272701 (NGS), SRR30272706 (ONT), SRR30272707 (Hi-C), SRR30272694-SRR30272700 (transcriptome), and SRR30272702-SRR30272705 (transcriptome). The BioSample is available with accession number SAMN43204218 at NCBI. The assembled genome is available at NCBI BioProject, the accession number is PRJNA1147874, and the genomes also are available in figshare 10.6084/m9.figshare.26772175. Supplementary materials are available at figshare: 10.6084/m9.figshare.26772169 (for gene annotation), 10.6084/m9.figshare.26772298 (for Repeat, RNA annotation, Interproscan analysis result and software and algorithms). Data are also available at GSA figshare https://doi.org/10.25387/g3.26772037. No specific scripts were used in this work. All commands and pipelines used in data processing were executed according to the manual and protocols of the corresponding bioinformatics software. Supplemental material available at G3 online.
